# Supplementary material for: A generalisation of the method of regression calibration and comparison with Bayesian and frequentist model averaging methods
Source: ArXiv. 2024 Mar 13:arXiv:2312.02215v3. Preprint. [Version 3] (PMC10775349)
Supplement: 1 [file NIHPP2312.02215V3-supplement-1.pdf]

Supplement A.

Table A1. Assumed distribution of persons by radiation dose group, based in part on distribution of person years in the Japanese atomic bomb survivor Life Span Study[1]

Dose group	Central estimate of dose (Gy)	Scaled numbers of persons
1	0.01	2591
2	0.1	334
3	0.5	438
4	1.5	102
5	2	6

References

1 Hsu, W.-L. *et al.* The incidence of leukemia, lymphoma and multiple myeloma among atomic bomb survivors: 1950-2001. *Radiat. Res.* **179**, 361-382 (2013). https://doi.org/10.1667/RR2892.1 [doi]
